# Serological screening for celiac disease in schoolchildren in Jordan. Is height and weight affected when seropositive?

**DOI:** 10.1186/1824-7288-36-16

**Published:** 2010-02-09

**Authors:** Mohamad K Nusier, Hedda Konstanse Brodtkorb, Siv Elisabeth Rein, Ahmed Odeh, Abdelrahman M Radaideh, Helge Klungland

**Affiliations:** 1 Department of Biochemistry and Molecular Biology, Faculty of Medicine, Jordan University of Science and Technology, Irbid 22110, Jordan; 2 Department of Laboratory Medicine, Children's and Women's Health, Faculty of Medicine, Norwegian University of Science and Technology, N-7006 Trondheim, Norway

## Abstract

**Background:**

Celiac disease (CD) emerged as a public health problem, and the disease prevalence varies among different races. The present study was designed to investigate the prevalence of CD using serological markers in apparently healthy schoolchildren in Irbid City, Jordan. Additionally, the effect of positive serology on height, weight and body mass index (BMI) was evaluated.

**Methods:**

The study population consisted of 1985 children (1117 girls and 868 boys), age range was 5.5 to 9.5 years. Height and weight were measured and blood samples were collected from each individual. Serum samples were analyzed for IgA anti-tissue transglutaminase antibodies (tTG) using a commercial enzyme-linked immunosorbent assay (ELISA). tTG positive samples were further analyzed for IgA anti-endomysium antibodies (EmA) with a commercial ELISA. Samples confirmed positive with EmA were considered seropositive.

**Results:**

Sixteen children were CD positive. The serological prevalence was estimated to be 1:124 (0.8%; 95% CI, 0.5% to 1.3%). Significant impact on growth (height) was found in seropositive children. When both sexes were individually analyzed, only boys showed height reduction. Furthermore, seropositive boys also had a significant weight reduction.

**Conclusion:**

This study demonstrated that CD is prevalent among schoolchildren in Jordan. The seropositive children tend to have lower height, weight, and BMI than the seronegative group. These differences were significant only for boys. None of the participants is known to have CD prior to the study.

## Background

Celiac disease (CD) is an immune-mediated small-bowel enteropathy. The disease is triggered by ingestion of gluten containing diet such as wheat, barley, and rye in genetically susceptible individuals [1]. Globally, the DQ2 type II human leukocyte antigen (HLA) is found in 90-95% of individuals suffering from CD. The remaining 5%, express the HLA-DQ8 variant [2]. The pathological features are a typical flat mucosa, abnormal surface epithelium, villous atrophy and hyperplastic crypts in the small intestine. The 1990 European Society for Pediatric Gastroenterology and Nutrition (ESPGAN) criteria for the diagnosis of CD require typical intestinal mucosa alterations [3]. Resolution of histological changes or symptoms is needed as a response to a strict gluten free diet (GFD) [4].

CD is a disease with a wide spectrum of manifestations, ranging from no apparent effect, to chronic diarrhea, abdominal distention, muscle wasting, hypotonia, poor appetite and distress (Table 1) [5]. Mortality and morbidity in CD patients are modestly increased compared to the general population [6]. Several studies have reported reduced growth in seropositives [7,8]. The only known cure for CD is a strict, lifelong adherence to GFD [9,10].

**Table 1:** Reasons for serological testing and grouping of celiac disease (CD) by symptoms (Fasano 2005, Rostom et al 2004).

	Classical CD	Atypical CD	Silent CD
Reasons for testing:	Investigation of intestinal symptoms.	Iron deficiency, osteoporosis, short stature, or infertility.	Screening.

Symptoms:	Intestinal symptoms.	Unusual intestinal complaints or extraintestinal manifestations.	Asymptomatic.

Serological screening is simple; it provides prevalence close to the biopsy proven CD prevalence. Serological evidence of CD has been reported to be 0.3-1.4% in population-based screening studies in a number of countries (Table 2) [7,11-14]. The prevalence of CD based on clinical symptoms has been estimated to be between 1: 1000 and 1: 10000 [10]. Serological screening studies have altered the perception of CD from a rare disorder, into a rather common condition.

**Table 2:** Results of different screening studies for celiac disease, CD

*Country*	*Age (years)*	*Number*	Antibody*	*Prevalence*	*Reference*
United Kingdom	7.5	5470	1. tTG 2. IgA EmA	1:101 (1.0%)†	(Bingley et al 2004)
The Netherlands	2 to 4	6127	1. IgA EmA	1:82 (1.2%)‡	(Csizmadia et al 1999)
The USA	2 to 18	1281	1. IgA EmA	1:320 (0.3%)§	(Fasano et al 2003)
Finland	7 to 16	3654	1. IgA EmA and IgA tTG	1:73 (1.4%)||	(Maki et al 2003)
Turkey	Adult blood donors	2 000	1. IgA tTG	1:87 (1.1%)‡	(Tatar et al 2004)
North America and Western Europe	Children	Large population	Biopsy	0.5% to 1.6%.	AHRQ No. 104 Celiac Disease 2004

tTG: tissue transglutaminase; EmA; anti-endomysium antibody.

*: 1. = the test(s) performed as screening test(s). 2. = the test performed for confirmation of the screening test.

† The prevalence is based on subjects confirmed positive with a second serological marker.

‡ The prevalence is based on subjects positive on only one serological marker.

§The prevalence is based on one serological marker (IgA EmA), but all tested positive on IgA tTG as well.

|| Two tests were carried out at the same time, and the serological prevalence is based on subjects positive on both tests.

Serological screening for CD has not yet been carried out in Jordan, and CD is not a common diagnosis among children in Jordan [15]. The aim of this study was to estimate the prevalence of CD using serological markers among a group of children aged 5.5 to 9.5 years in Irbid City, and to compare height, weight, and body mass index (BMI) in the seropositive group to the seronegative group.

## Methods

This is a collaborative-research study between the Norwegian University of Science and Technology (NTNU) and the Jordan University of Science and Technology (JUST).

Children in 20 elementary schools, grade one to four, in Irbid City, Jordan, were invited to participate in the study. Selection of schools was random. Eighteen schools were public and two were private. Both urban and rural areas were covered and socioeconomic status varied. None of the participants was reported to have CD. Twenty-four children 10 - 12 years old were excluded because they were too old. The study population consisted of 1985 children (1117 girls) age 5.5 - 9.5 years; mean, 8 years. According to the Registry and Statistical Department in Jordan, Irbid City had 54592 inhabitants between 6 and 10 years at the time of the study.

The study was approved by the Ministry of Health, the Ministry of Education in Jordan and the ethical committee of JUST. The study was performed according to the ethical standards for human research of the Helsinki Declaration [16]. Parents or legal guardians of participants were asked to provide a written consent.

Collection of blood samples, information and measurements were performed in schools between October 11^th ^and November 5^th ^2006. Information about age, sex, and a possible former CD diagnosis were also collected. All height and body weight measurements were performed by one researcher.

**Figure 1:**
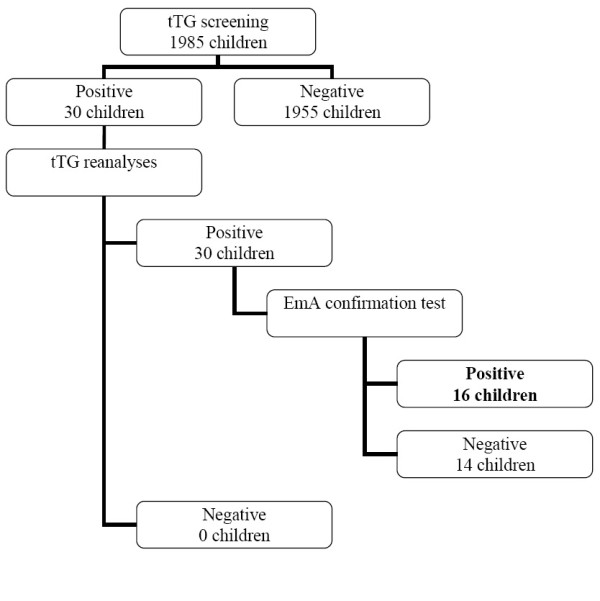
**Flowchart for procedure and results of serological screening for celiac disease among schoolchildren in Irbid city, Jordan**.

Serum samples were analyzed in duplicates for IgA antibodies to human tissue transglutaminase (tTG) using enzyme linked immunosorbent assay (ELISA) (Orgentec Diagnostika GmbH, Mainz, Germany). The test has a lower detection limit of 1.0 U/ml and 10 U/ml was the cut-off point for positive result. Positive samples for tTG antibodies were reanalyzed for conformation. Positive results were further analyzed with a commercial ELISA for IgA autoantibodies to human endomysial autoantigens (EmA) (GA Generic Assays GmbH, Dahlewitz, Germany). The analytical sensitivity of this test was 3 U/ml and the cut-off value was 20 U/ml. ELISA-tests were performed manually at King Abdullah University Hospital, JUST using an automatic washer (ELx 50 Auto Strip washer, Bio-Tek Instruments NC) and a Bio-Rad Eliza reader (680 Model), Bio-Rad Laboratories, UK. EmA positive subjects were defined as the seropositive group (n = 16), while the rest of the study population constituted the seronegative group (n = 1969) (Figure 1).

Subjects were divided into different age groups using half-year as accuracy level. Mean age and sex distribution were found in the whole study population and the serological prevalence of CD was calculated. The 95% confidence interval (95% CI) of the estimated prevalence was computed by the Agresti-Coull method:

x = number of seropositives. n = number in the study population.

Regression analysis was used to compare the measurements and BMI in the seropositive group with the values in the seronegative group. This approach gave age- and sex-adjusted estimates. The value of interest, for instance height, was entered as dependent variable. Covariates were age, sex, and seropositive group. The significance of interactions between the two covariates sex and seropositive group was tested for each value of interest. A separate regression analyses for each sex on each dependent variable was performed. With age and seropositive group as covariates, age-adjusted estimates were obtained. A two-sided *P *< 0.05 was considered significant. Analyses were performed using SPSS 13.0 for Windows.

## Results

Among the 1985 children, 16 were seropositive, of those 9 were girls; median age was 8 years and the age-range was 6 to 9.5 years (Figure 1). The serological prevalence of CD in this population is 1:124 (0.8%; 95% CI, 0.5% to 1.3%). The ratio between both sexes was equal in the study population and the seropositive group.

Seropositive group tends to have lower height, weight, and BMI than the seronegative group, but the difference was only significant for height (*P *= 0.027). The difference for BMI (*P *= 0.217) was not significant whereas for weight, but it was very close to significance (*P *= 0.055). There were no significant differences between both sexes, neither regarding height (*P *= 0.079), weight (*P *= 0.202), nor BMI (*P *= 0.502). The growth parameters in the seropositive boys were generally lower than those in the seropositive girls. The seropositive boys were significantly shorter (*P *= 0.014) and lighter (*P *= 0.015) than seronegative girls, but BMI values were not significantly lower (*P *= 0.078). Differences were not significant when comparing seropositive girls with seronegative girls, for height (*P *= 0.919), weight (*P *= 0.414) and BMI (*P *= 0.330).

## Discussion

The worldwide serological prevalence of CD ranges from 0.3% to 1.4% (Table 2) [7,11-14] and the CD prevalence of this study is in agreement with previous screening studies. Study-designs have shown variability in population studied, population size, testing procedures, definition of seropositive result, and which serological test the estimated prevalence is based on. These discrepancies contributed to the variability of reported prevalence. AHRQ reported a prevalence of CD in children by biopsy of 0.5% to 1.6% [17].

Our results as well as other studies have demonstrated that growth parameters are affected in CD seropositive group [7,8]. Discrepancies in study design and population, make it difficult to compare those findings with ours. In a study by Hoffenberg et al 2004, tTG-positive children have reduced z-scores for weight-for-height (-0.3 ± 0.7 vs. 0.3 ± 1; *P *= 0.02) and BMI (-0.3 ± 0.7 vs. 0.4 ± 0.9; *P *< 0.01) compared to controls, but did not show significant changes concerning weight- or height-for-age. Since two concordant serological markers were used to define the seropositive group in our study, it is likely to assume that a higher percentage of our seropositive group will be diagnosed with CD than in the study by Hoffenberg et al 2004.

The Agency for Healthcare Research and Quality (AHRQ) report found a prevalence of CD in children by biopsy of 0.5% to 1.6% and by serology of 0.3% to 1.9% [17]. Even though, the gold standard for the diagnosis of CD is duodenal biopsy, recent reports have shown that tTG alone is a sensitive marker for CD, yet noninvasive [18]. tTG is the marker of choice for CD mass screening and helpful in identifying patients who can benefit from gluten free diet and follow-up [19,20].

Another study used a two-step approach to define the seropositive group, found that seropositive children were lighter by 0.54 z-scores and shorter by more than 0.76 z-scores than seronegative controls [7]. Similarly, we found significant height reduction in the seropositives compared to controls. They also reported that seropositive children weighed significantly less than the controls. This also seems to be a trend in our study, although not significant (*P *= 0.055). Nonetheless, Bingley et al 2004, reported a study population of 5470 with 54 seropositive individuals. With a larger seropositive group, significant differences would more likely be present. Interestingly, the trend we see in weight reduction seems to be in accordance with the study by Bingley and coworkers.

The serology test results can be interpreted in different ways. We have chosen a two-step approach, where positive tTG was confirmed with positive EmA. Combining tests yield a higher positive predictive value (PPV) at the expense of missing some single marker positive patients [21]. If we consider only tTGA positivity as sufficient, the estimated prevalence in our study would rise to 1:66 (1.5%; 95% CI, 1.1% to 2.2%). This approach gives an increased sensitivity at the cost of specificity.

The positive predictive value (PPV) and negative predictive value (NPV) of a test result depend on the prevalence of the disease in the population tested. We performed screening on healthy children, where the prevalence of CD is low compared to a population with suspected CD. The PPV of a positive serology test drops as CD prevalence decreases [2]. As PPV falls, the risk of false positive CD patients increases. Because of the low prevalence of CD in our population, we required two concordant tests to improve PPV. NPV and CD prevalence demonstrate the reverse relationship as PPV and CD prevalence [2]. With a low CD prevalence in the population tested, NPV of the serological tests is high.

In a study, 31 of 57 children positive for IgA EmA had biopsies compatible with CD [11]. This means that the test's PPV was 54.4%. In the study by Hoffenberg et al 2004, the PPV of the tTG test was 72.2%. In another study the PPV was 82.9%, in the group which tested positive for both EmA and tTG [13]. This supports that PPV improves with two concordant tests.

Selective IgA deficiency is associated with CD patients [22]. CD patients with IgA deficiency do not express the IgA-type antibodies. A screening study among children found the prevalence of both CD and IgA deficiency to be 1:1218 [13]. This indicates that the combination is rare in asymptomatic children. In contrast, IgA deficiency occurs in about 2% of the symptomatic CD children [23]. Therefore, IgA deficiency must be considered in the assessment of symptomatic patients. According to Hill et al 2005, "the strategy of routinely determining serum IgA levels or adding IgG-based serology as part of a panel to screen asymptomatic individuals in the general population is not warranted." We may, however, have missed CD patients with IgA deficiency [24].

Our serological prevalence is a close estimate of CD in the population. The study may have false positive and false negative CD cases because the correlation between serological markers and small bowel biopsy is not 100%. Furthermore, false seropositives may represent a source of error when comparing growth parameters.

Even though, screening may detect silent CD, clinical benefits of identifying all cases of CD remain controversial [8]. To justify population-based screening, advantages should outweigh the disadvantages of being diagnosed with CD. A screening study performed in Italy found that two thirds of the biopsy proven CD were asymptomatic [25].

Our study has shown that CD is a common disorder in Jordan and physicians must consider CD in patients presenting a wide range of clinical symptoms.

## Conclusion

A serological prevalence of 1:124 (0.8%) indicates that CD is a common disorder among schoolchildren in Jordan. It demonstrates that Jordanian physician should have a high index of suspicion for CD. The serological prevalence found in this study is in accordance with the results of similar studies. In the seropositive group a tendency to lower height, weight, and BMI than in the seronegative group was found, but the difference was only significant for height. The seropositive boys tended to be more growth-affected than the seropositive girls, but the differences between the sexes were not significant. This study underscores that Jordanian physician should have a low threshold for suspecting CD.

## Competing interests

The authors declare that they have no competing interests.

## Authors' contributions

MN carried out the design and supervision of the study and manuscript writing and editing. HB and SR performed the field and laboratory studies and data analysis. AO and AR performed clinical support and consultation. HK carried out study supervision and statistical analysis. All authors have read and approved the final manuscript.
